# Attitudes towards lung cancer screening in socioeconomically deprived and heavy smoking communities: informing screening communication

**DOI:** 10.1111/hex.12481

**Published:** 2016-07-11

**Authors:** Samantha L. Quaife, Laura A. V. Marlow, Andy McEwen, Samuel M. Janes, Jane Wardle

**Affiliations:** ^1^ Health Behaviour Research Centre Department of Epidemiology and Public Health University College London London UK; ^2^ Lungs for Living Research Centre UCL Respiratory Division of Medicine University College London London UK

**Keywords:** inequalities, lung cancer, screening, smoking, socioeconomic status

## Abstract

**Background:**

While discussion continues over the future implementation of lung cancer screening, low participation from higher risk groups could limit the effectiveness of any national screening programme.

**Objectives:**

To compare smokers’ beliefs about lung cancer screening with those of former and never smokers within a low socioeconomic status (SES) sample, to explore the views of lower SES smokers and ex‐smokers in‐depth, and to provide insights into effective engagement strategies.

**Design, setting and participants:**

Using proactive, community‐based recruitment methods, we surveyed 175 individuals from socioeconomically deprived communities with high smoking prevalence and subsequently interviewed 21 smokers and ex‐smokers. Participants were approached in community settings or responded to a mail‐out from their housing association.

**Results:**

Interviewees were supportive of screening in principle, but many were doubtful about its ability to deliver long‐term survival benefit for their generation of “heavy smokers.” Lung cancer was perceived as an uncontrollable disease, and the survey data showed that fatalism, worry and perceived risk of lung cancer were particularly high among smokers compared with non‐smokers. Perceived blame and stigma around lung cancer as a self‐inflicted smokers’ disease were implicated by interviewees as important social deterrents of screening participation. The belief that lungs are not a treatable organ appeared to be a common lay explanation for poor survival and undermined the potential value of screening.

**Conclusions:**

Attitudes towards screening among this high‐risk group are complex. Invitation strategies need to be carefully devised to achieve equitable participation in screening.

## 1 Introduction

In 2011, there were over 35 000 deaths from lung cancer in the UK, representing 22% of total cancer mortality.^1^ Early stage at diagnosis is the strongest predictor of survival^2^ but only 15% of patients are diagnosed at Stage 1,^3^ and the diagnosis is often incidental.^4^ One promising strategy for improving outcomes is to screen for early stage disease in high‐risk smokers and ex‐smokers using low‐dose computed tomography (LDCT). The National Lung Screening Trial (NLST) found a 20% relative risk reduction in lung cancer mortality following three annual LDCT screens compared with chest X‐ray,^5^ and the United States Preventive Service Task Force (USPSTF) recommend screening in conjunction with smoking cessation counselling.^6^ However, a favourable harm–benefit screening ratio depends on engaging those at high risk with screening.^7^, ^8^

Individual risk prediction is becoming increasingly sophisticated and the science of identifying eligible screening candidates ever more precise.^9^ Smoking is the key risk factor for lung cancer, accounting for 86% of diagnoses made within the United Kingdom;^10^ with other risk factors including occupational exposures,^11^ respiratory disease^12^ and a family history of lung cancer.^13^ Rates of smoking in the UK are highest in socioeconomically deprived areas, where lung cancer incidence and survival are worse.^14^ Lower socioeconomic status (SES) may amplify risk due to younger age of starting smoking, and greater tobacco smoke inhalation, nicotine dependence, second‐hand smoke exposure and difficulty quitting.^15^, ^16^, ^17^, ^18^ Other risk factors, such as a history of pneumonia, also increase with measures of deprivation.^19^ In the UK Lung Screening Trial (UKLS), the number of participants registering a high score on the Liverpool Lung Project (LLP) risk prediction model ranged from 8% to 18% from the least to most socioeconomically deprived quintiles.^20^

Smokers from deprived communities are therefore an important group to engage with screening, but participation in trials has been skewed towards former smokers and the better educated.^20^, ^21^ Compared with an eligible population cohort (a subsample of respondents to the US Census Department's Tobacco Use Supplement), NLST participants were younger, had more years of education and were more likely to be former (than current) smokers.^22^ Surveys carried out away from clinical settings in the United States have begun to identify attitudes that might discourage smokers’ participation. One survey found smokers were less likely to perceive survival benefit from lung cancer screening or believe they would undergo surgery for a screen‐detected cancer.^23^ In an ethnically diverse sample, fatalism, concern about radiation and anxiety about having a CT scan predicted lower screening intentions.^24^

Three studies^25^, ^26^, ^27^ specifically investigated the attitudes of those declining screening offered in the trial context, although the response rate in this group (of all those invited) is usually <10%. Non‐participants who responded to a follow‐up survey (n=97) in the Dutch–Belgian trial NELSON were found to perceive screening as too much effort or unnecessary due to a lack of respiratory symptoms.^25^ In the UK Lung Screening Trial (UKLS), a survey of non‐participants (n=748) found a small proportion of smokers to report emotional barriers to participation, but practical barriers were most commonly cited.^26^ A qualitative study of patients (n=24) declining participation in a UK screening trial (Lung‐SEARCH) for COPD patients identified four types of attitudes explaining reluctance to participate: worry, fatalism, avoidance and believing oneself too old to benefit.^27^

There are no data from the United Kingdom on beliefs about lung cancer screening among individuals from socioeconomically deprived communities with high smoking prevalence, a group less likely to engage in research carried out through traditional channels and for whom proactive recruitment methods may be better suited. The effectiveness of any lung cancer screening programme depends, in part, upon uptake and any inequalities in participation ultimately have the potential to exacerbate inequalities in lung cancer survival. Understanding why screening participation is low among this high‐risk group would contribute to the development of evidence‐based engagement strategies and invitation materials to ensure the reach of any future screening programme is equitable. Therefore, this study investigated attitudes towards lung cancer screening within lower SES communities with the aim of (i) understanding how attitudes might differ by smoking status, (ii) exploring the attitudes of smokers and ex‐smokers in‐depth and (iii) identifying factors that could be targeted in screening communication strategies.

## 2 Method

### 2.1 Mixed methods design

Study one used a quantitative survey design to compare smokers’ beliefs about lung cancer screening with those of former and never smokers, recruited from lower SES communities. Study two interviewed a subset of survey participants to explore the views of smokers and former smokers in‐depth, to investigate factors that might be important for screening participation. Ethics approval was granted by the UCL research ethics committee (reference: 5210/001).

### 2.2 Recruitment methods

Proactive, community‐based strategies were used to recruit individuals from lower SES communities, with the aim of involving individuals in research who might be less likely to participate in research carried out using traditional recruitment methods, central to understanding non‐participation. Individual people leading activities, organizations, venues or development work in socioeconomically deprived areas were identified from online searches, council listings and literature displayed in community centres. Selections were made on the basis that they (i) had networks with relevant local groups; (ii) provided free support and outreach services to those living in deprived conditions; (iii) worked within a manual workplace where smoking rates are higher; (iv) provided social housing; and (v) were not providing a health service. A researcher (SLQ) met with interested community leaders to seek advice on recruitment approaches and to access their networks to identify other recruitment opportunities. The recruitment approach was two‐pronged for both studies: (i) participants were approached directly in community settings by SLQ and (ii) two housing associations mailed the survey to tenants. Recruitment settings ranged from organized community‐based services (e.g. free drop‐in advice service for housing issues) to locations with a high footfall (e.g. bus station and markets).

### 2.3 Survey Study one

#### 2.3.1 Participants

Adults aged ≥40 years were eligible. Adults younger than the screening‐eligible age (aged 55–80 years^6^) were included because they are approaching eligibility and are representative of the generation who would comprise the first screening cohort in the United Kingdom, pending recommendation of screening. Never smokers were included for comparative purposes, because former smokers were once current smokers and may share characteristics associated with smoking uptake. The inclusion of never smokers also intended to provide insight into the wider social context within which screening would be considered.

Participants were recruited from lower SES communities in Central and South‐East London. As an example, in one of the South‐East London boroughs, population statistics indicate that 86% of residents are classified within the two most deprived Index of Multiple Deprivation (IMD) quintiles.^28^ Smoking prevalence is highest within these quintiles: 33% of men and 26% of women smoke in the most deprived IMD quintile, and 26% of men and 20% of women smoke in the second most deprived IMD quintile.^29^

#### 2.3.2 Measures

A survey was constructed using items adapted from existing measures of cancer beliefs (for lung and other types^24^, ^30^, ^31^, ^32^, ^33^, ^34^), and original items were developed based on the findings of an existing qualitative study.^27^ The inclusion of items was therefore driven by the available literature rather than a specific theoretical model.

The survey began with a brief description of screening, which explained that the test aimed to find lung cancer at an early stage when there was a better chance of cure, and that it used a “type of X‐ray called a CT scan.” Participants were then asked to rate their agreement (strongly disagree/disagree/agree/strongly agree/don't know) with each item. Items covered the following topics in relation to lung cancer or lung cancer screening: smoking, worry and avoidance, perceived benefit, lung cancer outcomes, perceived risk, practical barriers and the need for screening without symptoms.

Smoking data were also collected, including self‐reported smoking status, age started daily smoking and maximum number of cigarettes smoked daily (to calculate pack‐year history), number of quit attempts lasting ≥3 months, age of quitting and quit confidence (on a scale of 0 to 10). Demographic characteristics included sex, age, marital status, ethnicity, education, postcode (to calculate IMD rank^35^) and employment status.

The survey was paper‐based and designed to be self‐completed. SLQ assisted participants with completing the survey if they had difficulty or preferred it to be read aloud.

#### 2.3.3 Analyses

Univariate chi‐square analyses and Fisher's exact tests were carried out to explore associations between smoking status and item agreement. Multivariate analyses were not carried out because there were too few cases to provide adequate statistical power. For the same reason, we did not lower the statistical significance threshold to adjust for multiple testing, but report the respective significance thresholds.

### 2.4 Interview Study two

#### 2.4.1 Participants

After each participant had completed the survey, the researcher screened their responses to purposively sample a subgroup of interviewees who had indicated they were current or former smokers, and from lower SES backgrounds (as indicated by an area‐based measure of deprivation or their education level). Arrangements for interview were made immediately with participants who indicated they were willing to be interviewed, to minimize attrition. Therefore, recruitment for interviewing and the survey began at the same time and interviews ceased when no new information was gleaned from the data.

#### 2.4.2 Measures

Semi‐structured telephone interviews were carried out, during which participants were asked open‐ended questions on three main topics: (i) benefits/barriers to screening (e.g. “Can you talk me through your reasons for going/not going [to screening]?”), (ii) the preferred approach for screening invitations (e.g. “Often, letters from the doctor point out that the person is a smoker. How do you feel about that?”) and (iii) attitudes towards provision of smoking cessation support (e.g. “Smokers may also be asked if they would like advice or help with stopping smoking at their appointment. How do you feel about that?”). At the start of the interview, the same brief explanation of screening was given as in Study one, but participants were also asked to imagine they had received a screening invitation from their GP.

#### 2.4.3 Analyses

Transcripts were analysed using an inductive approach to thematic analysis with NVivo qualitative data analysis software (QSR International Pty Ltd. Version 10, 2012). Familiarization with the data began during the interviews and continued through repeated reading of the transcripts. Data were first coded openly, with minimal interpretation. Themes and sub‐themes were then interpreted and developed into a framework. Inclusion of themes depended on their frequency and contribution to the research question, that is factors that may be important when communicating a screening offer. A second researcher (LAVM) read ten randomly selected transcripts independently, to cross‐check the framework, and any disagreements were resolved through discussion and revisiting the transcripts.

## 3 Results

### 3.1 Sample characteristics

Most participants were approached directly in the community context by SLQ (n=14 interviewees; n=100 questionnaire respondents). The remaining number responded to a questionnaire mail‐out from two housing associations (n=7 interviewees; n=75 respondents). Around three‐quarters of individuals approached in person completed the survey and the majority of eligible participants (>90%) subsequently contacted for interview took part. The response rate for mailed surveys was very low (12%). Post‐hoc analyses showed that participants recruited in‐person were more frequently younger and current smokers (*P*<.01) and had a higher level of education (*P*<.05) than those who responded to the mail‐outs. There were no differences by deprivation, sex, ethnicity or marital status.

In total, 175 participants completed the survey. Occasional smokers (n=7) and those reporting a diagnosis of lung cancer (n=5) were excluded from analyses. Of the remaining 163 participants, 28% identified as current smokers, 44% were ex‐smokers and 29% had never smoked. Of these, nine smokers and 12 ex‐smokers (N=21) were interviewed (see Table 1).

**Table 1:** Demographic and smoking characteristics

	Interview (n=21)	Survey (n=163)
Sex, % (n)		
Male	52.4 (11)	41.1 (67)
Female	47.6 (10)	58.9 (96)
Age group, % (n)		
41–49	4.8 (1)	5.5 (9)
50–59	33.3 (7)	24.5 (40)
60–69	52.3 (11)	33.1 (54)
70–79	9.5 (2)	27.6 (45)
80+	0 (0)	3.1 (5)
Marital status, % (n)		
Married/Civil partnership/Cohabiting	14.3 (3)	33.7 (55)
Single/Divorced/Widowed	85.7 (18)	66.3 (108)
Ethnicity, % (n)		
White British/Irish/White other	90.5 (19)	78.5 (128)
Not White	9.5 (2)	19.6 (32)
Highest level of education, % (n)		
Left school at or before age 15/no formal qualifications	71.4 (15)	54.6 (89)
CSEs/O‐levels/ONC/BTEC/Other	19.0 (4)	23.3 (38)
A‐levels/Higher education qualification below degree	4.8 (1)	9.2 (15)
University degree	4.8 (1)	12.9 (21)
IMD rank quintile (rank range), % (n)		
Quintile 1 (1–6496) most deprived	76.2 (16)	50.9 (83)
Quintile 2 (6497–12993)	14.3 (3)	26.4 (43)
Quintile 3 (12994–19489)	4.8 (1)	1.2 (2)
Quintile 4 (19490–25986)	0 (0)	3.1 (5)
Quintile 5 (25987–32482) least deprived	0 (0)	0.6 (1)
Employment status, % (n)		
Employed full time/Part time/Self employed	23.8 (5)	28.8 (47)
Unemployed	14.3 (3)	8.6 (14)
Full‐time homemaker/Carer	0 (0)	4.3 (7)
Retired	52.4 (11)	52.1 (85)
Disabled or too ill to work	9.5 (2)	5.5 (9)
Studying	0 (0)	0.6 (1)
Smoking status, % (n)		
Current smoker	42.9 (9)	27.6 (45)
Former smoker	57.1 (12)	43.6 (71)
Never smoker	0 (0)	28.8 (47)
Experience of lung cancer, % (n)		
Yes (someone close or prefer not to say who)	47.6 (10)	36.9 (60)
None	47.6 (10)	62.0 (101)
Smoking history, range (mean, SD)		
Mean age started smoking daily (≥1 cigarette or roll up)	6–39 (14.5, 6.5)	6–40 (16.7, 5.4)
Number of times stopped smoking (≥3 months)	0–6 (1.1, 1.7)	0–10 (1.1, 1.9)
Age stopped smoking (former smokers only)	38–66 (54.8, 9.2)	20–82 (50.1, 13.4)
Pack years	6–285 (67.6, 57.5)	0.5–285 (51.6, 43.5)
Quit confidence rating of 10 (current smokers only)	0–10 (4.4, 4.5)	0–10 (4.9, 3.5)

N and % totals may not sum due to missing data.

Men and women were evenly represented. Samples had a similar mean age (interviews: 62 years; survey: 64 years) and were mostly white (91% and 79%), unmarried or widowed (86% and 66%) and retired (both 52%). The majority were from lower SES backgrounds, as indexed by an area‐based measure of deprivation (most deprived IMD quintile: 76% and 51%), and education (no formal qualifications/left school age ≤15 years: 71% and 55%). The higher proportion of lower SES interviewees compared with survey respondents was intentional and part of the purposive sampling frame. Many participants reported that they had experience of lung cancer through a “close other” (48% and 37%).

With regard to smoking history, on average, current and former smokers had begun smoking in their mid‐teens (M: 14.5; SD: 6.5 and M: 16.7; SD: 5.4) and accrued a high pack‐year history (M: 67.6; range: 6–285 and M: 51.6; range: 0.5–285; eligibility for screening is ≥30 pack‐years^6^). Current smokers were largely not confident in their ability to quit (mean rating of 4.4 out of 10; SD: 4.5 and M: 4.9; SD: 3.5).

### 3.2 Univariate analysis of survey data

The key findings are described here, but all univariate results are shown in Table 2 and Table S1.

**Table 2:** Frequencies, chi‐square analyses and Fisher's exact tests for agreement with each belief item by smoking status (Survey Study one)

	Smoking status % (n) agree				
	Overall (n=163)	Current (n=45)	Former (n=71)	Never (n=47)	Sig.
Smoking					
The people doing the lung cancer screening could be rude to smokers	13.1 (21)	20.5 (9)	8.7 (6)	12.8 (6)	.489*
There is no point going for lung cancer screening while you are still smoking	10.7 (17)	2.3 (1)	14.5 (10)	13.0 (6)	.046**
If the CT scan is negative, you can continue to smoke without worrying about lung cancer	12.0 (19)	29.5 (13)	5.8 (4)	4.4 (2)	<.001**
I have smoked too long to benefit from lung cancer screening	10.3 (12)	20.0 (9)	4.3 (3)	–	.020**
Perceived risk					
My personal risk of getting lung cancer during my lifetime is higher than other smokers	–	35.6 (16)	–	–	–
I would have got lung cancer by now if I was going to	8.4 (13)	9.1 (4)	5.9 (4)	11.9 (5)	.505**
I think I have a high chance of getting lung cancer in the next few years	19.5 (31)	47.7 (21)	10.1 (7)	6.5 (3)	<.001**
I think I may already have lung cancer	8.2 (13)	17.8 (8)	4.3 (3)	4.5 (2)	.053**
There's no risk of getting lung cancer if you only smoke for a few years	5.0 (8)	4.4 (2)	7.2 (5)	2.2 (1)	.534**
I feel I will get lung cancer sometime during my life	21.7 (35)	44.4 (20)	10.1 (7)	17.0 (8)	<.001**
Once you stop smoking you are no longer at risk of lung cancer	8.8 (14)	8.9 (4)	11.6 (8)	4.4 (2)	.264**
Worry					
A clear CT scan would stop me worrying about lung cancer	69.7 (108)	54.5 (24)	80.6 (54)	68.2 (30)	.023*
I often worry about my chance of getting lung cancer	38.0 (62)	75.0 (33)	24.6 (17)	26.7 (12)	<.001**
I'd be too worried about lung cancer to have a lung cancer screening test	11.0 (18)	13.3 (6)	11.3 (8)	8.5 (4)	.095**
I'm very scared of getting lung cancer	57.8 (93)	60.0 (27)	54.3 (38)	60.9 (28)	.426**
Lung cancer outcomes					
If I ever got lung cancer, I could be cured***	38.8 (47)	28.0 (7)	36.8 (21)	48.7 (19)	.125**
A diagnosis of lung cancer is a death sentence	22.0 (35)	47.7 (21)	13.0 (9)	10.9 (5)	<.001*
Lung cancer can often be cured	46.3 (74)	40.9 (18)	50.7 (35)	44.7 (21)	.498*
These days many people with lung cancer can expect to continue with their normal activities and responsibilities	44.9 (71)	39.5 (17)	54.3 (38)	35.6 (16)	.085*
Most lung cancer treatment is worse than lung cancer itself	21.6 (35)	20.5 (9)	19.7 (14)	25.5 (12)	.713*

*Chi‐square analyses, **Fisher's exact test. ***n=122.

### 3.3 Beliefs about smoking and lung cancer screening

Current smokers were most likely to agree with some of the beliefs about smoking which conflict with participating in or benefiting from screening. Twenty per cent (n=9) believed they had “smoked too long to benefit*”* (vs 4%, n=3 of former smokers; *P*<.05) and almost a third (30%, n=13) agreed that “if the CT scan is negative you can continue to smoke without worrying about lung cancer” (vs 6%, n=4 of former smokers and 4%, n=2 of never smokers; *P*<.001; see Table 2).

### 3.4 Perceived risk of lung cancer

More current smokers perceived their risk of getting lung cancer as high over “the next few years” (48%, n=21) than former (10%, n=7) and never smokers (7%, n=3; *P*<.001), and over a third (36%, n=16) agreed their lifetime risk “is higher than other smokers.” Forty‐four per cent (n=20) felt “I will get lung cancer sometime during my life” (vs 10%, n=7 and 17%, n=8, respectively, *P*<.001). Eighteen per cent (n=8) of smokers thought they “may already have lung cancer” but the proportion agreeing was not significantly different to former or never smokers (see Table 2).

### 3.5 Worry about lung cancer

Three‐quarters (75%, n=33) of current smokers agreed they “often worry” about getting lung cancer (vs 25%, n=17 and 27%, n=12 of former and never smokers; *P*<.001), and fewer (55%, n=24) believed “a clear CT scan would stop me worrying” (vs 81%, n=54 and 68%, n=30; *P*<.05). However, smokers were no more likely to agree worry would deter them from screening or that they were “very scared of getting lung cancer”, items endorsed across smoking groups (see Table 2).

### 3.6 Lung cancer outcomes

Fewer than half (n=71) of the sample agreed “people with lung cancer can expect to continue with their normal activities” and over a fifth (22%, n=35) thought “treatment is worse than the lung cancer itself.” Smokers were most pessimistic about survival, with almost half agreeing lung cancer is “a death sentence” (48%, n=21 vs 13%, n=9 of former smokers and 11%, n=5 of never smokers, *P*<.001; see Table 2). However, there was no significant association with smoking status for agreeing that “lung cancer can often be cured.”

### 3.7 Online supplementary results

Additional results on beliefs about perceived benefits of screening, avoidance, symptoms, and practical barriers are provided in Table S1 in the interests of space. Briefly, there were almost no significant associations with smoking status. Overall, endorsement of perceived benefits was high (>64%), avoidant beliefs were held by around one‐fifth (18%–25%) and few endorsed practical barriers.

### 3.8 Thematic analysis of interview data

Themes comprise two categories: the first encompasses general attitudes to lung cancer and the second concerns screening‐specific attitudes. Short illustrative quotes are included in the text and longer quotes in Tables 3 and 4. Reference codes represent participant number (P), sex (F/M), smoking status (X/S = ex‐smoker/smoker) and age.

**Table 3:** Quotes illustrative of the general attitude themes (Interview Study two)

	Participant
Smoking: history, stigma and identity	
“I feel a bit ashamed I suppose… I don't like to say I smoke… it could be a bit of guilt really”	P11, F, S, 69
“So I'm on roll‐ups now… I've tried to give up, but I can't… I've had advice from my doctor and everybody. And I still can't… I wish I could”	P10, F, S, 63
“you don't enjoy it you get stuck in, you don't get much choice it's addictive”	P1, M, X, 54
“when you go back 40 years ago they didn't know nothing about it really, about cigarettes, the dangers of cigarettes, smoking and stuff”	P4, F, X, 58
“I lie to people… whatever's wrong with you they say it's down to smoking, pack it up and see ya later… you ain't gonna get the proper diagnosis”	P3, M, S, 57
“I think some of these doctors and ambulance men now are taking it a bit personal with people… Because I had one doctor… the other two, it was like taboo. They pushed me into a corridor… I think they were offish in the attitude what the people that think the result is it's self‐inflicted”	P12, M, S, 68
“I know that the doctor would tell me off about it… It would be your fault, actually.”	P17, F, S, 61
“people have been smoking since Robinson Crusoe… It's enjoyment for people, it's a working class man's thing… I'm no different to anybody else” *but later adds*, “if they could give me something to stop me smoking, I'd take it straight away”	P5, M, S, 56
Uncontrollable disease: risk, fatalism and treatment	
“I think you'll always be worried *[about lung cancer]* if you've been a heavy smoker”	P13, F, X, 66
“But I breathe heavy. I've got all the signs of lung cancer”	P10, F, S, 63
“my nan died of it, er one, two of my uncles died of it… so right the way back to the beginning of time right up until now. It's in the genes”	P5, M, S, 56
“I worked in a car plant… everything was covered in powder so you were breathing that… eight hours a day, so that is a concern as well.”	P9, M, X, 50
“they think, “I'll pack up smoking and live another ten years.” But life don't work that way, does it?… when your numbers up, your numbers up”	P12, M, S, 68
“I think it's [*lung cancer*] the luck of the draw isn't it, really, with everything”	P11, F, S, 69
“*[lung cancer]* slowly suffocates you, it's irreversible and they can't do nothing for it”	P5, M, S, 56
“it will kill you for sure.. I just know that you get cancer and you are dead”	P16, F, X, 67
“I think it [*chemotherapy*] knocks them off quicker in some cases… I would decline it, I would just take my chances”	P22, F, X, 67
“with breast cancer you can have your breast off, but in the lungs it's a very, I don't know, it seems a different kettle of fish to me”	P11, F, S, 69
Life circumstances: poor health, life experiences and addiction	
“I've known two or three people with it [*lung cancer*]… But the ones that I've known have not lasted that long… it's a bit frightening”	P11, F, S, 69
“She screamed in pain until she died for more than a week and it's a horrible way to go because it's very painful, very nasty”	P9, M, X, 50
“Everything always turns out bad or worse for me… Because I've had a lot of negative things in my life… Being brought up on a council estate”	P18, M, S, 47
“I'd fix myself with booze, drinks, drugs for years”	P18, M, S, 67
“I always thought I'd got lung cancer because I abused myself… Really badly abused myself. After I lost my mother and father I gave up”	P18, M, S, 47

**Table 4:** Quotes illustrative of the screening‐specific attitude themes (Interview Study two)

	Participant
Support screening: early detection, preparation, and reassurance	
“a cure could be round the corner, and if you're diagnosed early enough and being able to be sort of er put on hold… a cure might appear”	P2, M, X, 63
“what I've heard from TV. I mean, as far as I'm aware is if you catch it early enough you can survive… whether that's true, I'm not too sure”	P7, F, X, 57
“I'd prefer to know, you know ‘cause you could kind of get your affairs in order… I've got a big large, I've got five children… five grandchildren”	P4, F, X, 58
“if they can do me a favour and get me some extra years for those grandkids… I might even be lucky enough to… walk them down the aisle”	P12, M, S, 68
“Oh, it would be great for me *[negative result]*, and probably I would try my best not to do it, not to smoke”	P17, F, S, 61
“It's like a relief; it's something else off your shoulders, isn't it… Because you carry all this *[expletive]* about with you, thinking this, thinking that, and it's all negative stuff. And then you get to the truth *[a negative result]* and it's like ah! It's like a real buzz”	P18, M, S, 47
Fear: diagnosis, hospitals, getting sick and death	
“I'm just frightened because I smoke a lot. I've been smoking since I was ten years of age… I think the shock [*of a positive screen*] would kill me”	P10, F, S, 63
“they're maybe scared of hospitals, scared of dying… because a lot of people think, well, they go in with one thing and they catch all sorts”	P19, F, S, 65
“he'd just literally be too scared. He hates hospitals… I know he's had bad experiences… And that's his attitude: “If I go in there I won't come out again”	P7, F, X, 57
Avoidance: rather not know and wait until sick	
“I'd rather not know, you know, personally, and carry on as things are carrying on”	P12, M, S, 68
“He had a pain in his lung when he was coughing and he never went anywhere… and when he went to the doctors… he was dead within three weeks”	P5, M, S, 56
“They'd be frightened of knowing… my husband, he wouldn't dream of going to a doctor… He would rather not know. He'd rather just, you know, one day he's here, next day he's gone… I said to him, “The only time you'll see a doctor is when you're, you know, halfway upstairs or downstairs”… I think he would have to be on death's door before he actually sees the doctor”	P7, F, X, 57
Too late: smoking damage and older age	
“I mean it doesn't really bother me now, ‘cause I think it's too late anyway, so I reckon the damage is already done anyway”	P3, M, S, 63
“Like you know in any campaign they're running, yes the health benefits are great but believe me once you've smoked more than ten years you think if I've got it I've got it, there's nothing I can do about it”	P4, F, X, 58
“I'd go now. I'd be scared to go. But I would go if I was younger, you know… at my age I can't imagine me surviving”	P10, F, S, 63
“I would say from 25 onwards, to be honest… the younger, the better… they stand a chance to fight it. But with older people I don't know so much… I think that the younger persons… their body's younger, their organs are more… they are younger and healthier than an old person”	P22, F, X, 67
Screening: approach and support	
“if they think someone is going to be there wagging the finger at them, that's it, it's out the window…”If you point at people and say, “Look, you've been picked out and we think you ought to be screened,” that will frighten them to death”	P23, M, X, 66
“it's the way it's worded as well because a lot of people sort of go “oh” and panic”	P8, M, S, 52
“Well, when you get this invitation you are thinking, oh I wonder if I've got it, that's why they've sent it to me”	P16, F, X, 67

### 3.9 General attitudes

#### 3.9.1 Smoking: history, stigma and identity

Participants often explained or justified their smoking history, with many expressing “regret” and “guilt.” Current smokers in particular emphasized the difficulties of stopping, feeling “trapped” by addiction, and their attempts at cutting down. Some pointed out that when their generation began smoking, it was seen as “glamorous” and the risks were not well publicized.

Smoking was commonly described as a “stigmatized” and “taboo” behaviour, for which smokers had been “singled out” and “picked on.” Current smokers especially felt health professionals blamed them for their ill health and treated them unfairly, worrying that “you ain't gonna get the proper diagnosis” (P3, M, S, 57). This was especially true of interviewees who emphasized smoking as part of their identity (“it's a working class man's thing,” P5, M, S, 56) and attempted to normalize the habit as something going on “since Robinson Crusoe” (P5, M, S, 56). Tellingly, the same participant added: “if they could give me something to stop me smoking, I'd take it straight away” (P5, M, S, 56) (see Table 3).

#### 3.9.2 Uncontrollable disease: risk, survival and treatment

Lung cancer was described as a “killer” disease, which is “aggressive” and a “painful way to go” (P9, M, X, 50) by both current and former smokers. The vital nature of lungs was implicated in poor survival with some questioning how treatment is possible given that “you can't live without your lungs” (P9, M, X, 50). There was concern that treatment is ineffective and detrimental to quality of life. Some participants explained they would decline treatment, perceiving screening as something which “can never give you a new pair of lungs” (P1, M, X, 54).

Regardless of smoking status, many were concerned that there was little they could do to reduce their high risk of lung cancer, because of their smoking history. Some worried they may already have it: “you'll always be worried if you've been a heavy smoker” (P13, F, X, 66). To compound this lack of control, risk was perceived as unpredictable. Interviewees cited the fact that a non‐smoker can get lung cancer even if “they've never put a fag in their mouth” (P5, M, S, 56), and that “a lot of people smoke and still live to a ripe old age” (P16, F, X, 67). Suggestions of other risk factors further reduced perceived control, including genetics, pollution, asbestos, poor housing, workplace exposures, stress and “cancer‐grown foods” (P9, M, X, 50). Not smoking was therefore not necessarily perceived as protective and many attributed their risk to chance. A minority underplayed smoking's role in lung cancer risk as something that “doesn't help” (P7, F, X, 57), or “a load of old toffee” (P5, M, S, 56) (see Table 3).

#### 3.9.3 Life circumstances: poor health, life experiences and addiction

Poor health and comorbidities were commonplace among participants’ family and friends, which included other cancers, smoking‐related diseases (especially COPD) and chronic diseases (e.g. diabetes). Vicarious experiences of others’ suffering with lung cancer were commonly referenced in relation to screening attitudes. Difficult life circumstances beyond health were also mentioned and implicated by some in a pessimistic outlook on life. Some interviewees alluded to their struggles with other addictions (see Table 3).

### 3.10 Screening‐specific attitudes

#### 3.10.1 Support for screening: early detection, reassurance and preparation before death

Participants were positive in principle about screening, and several indicated that they would participate; often because they perceived benefit in knowing whether they have lung cancer. Early detection was most commonly cited with regard to “less major treatments” (P1, M, X, 54), “better chance of cure” (P14, M, X, 73) and leading “a normal life for longer” (P9, M, X, 50), but notably more frequently by former smokers than current smokers. Furthermore, the language used to convey early detection principles was typically cautious about the ability to survive lung cancer in the long‐term. Some appeared to pay “lip service” to the idea without real conviction, doubting the benefits: “whether that's true, I'm not too sure” (P7, F, X, 57). Instead, some participants perceived the benefit of early detection to be “halting the growth” (P23, M, X, 66) until a cure became available, indicating a lack of awareness that early stage treatment can be curative.

Some participants welcomed the potential for reassurance that that they did not have lung cancer and motivation to stop smoking if given a “clean slate.” Others saw screening as an opportunity to prepare family and personal affairs before they died. The importance of family was also apparent in the reasoning that “everyone wants to be around for their families” (P13, F, X, 66). One smoker began the interview resolutely against screening, but thought differently when considering his grandchildren (see Table 4).

#### 3.10.2 Fear: diagnosis, hospitals, getting sick and death

Fear of diagnosis was commonly suggested as a deterrent to screening due to the expectation of a positive result, particularly for current smokers. For some, the decision to attend was interpreted as a decision to “find out” (P4, F, X, 58) about their lung cancer. This fear also appeared to originate from concerns around the whole care pathway, including getting sick, hospitals as a slippery slope from which the sick do not return and ultimately death.

Some participants explained how receiving the invitation would frighten them, describing responses such as feeling “horrified,” “panic” and “I would never stop worrying until I get the result” (P16, F, X, 67). Participants were not concerned about the CT scan, which was generally regarded as “only like taking a picture of your insides” (P7, F, X, 57). The exception was one participant concerned about radiation. Following a previous CT scan, he believed he “was radioactive for the rest of my life” (P1, M, X, 54) (see Table 4).

#### 3.10.3 Avoidance: rather not know and wait until sick

Not wanting to know was described as a reason to avoid screening, especially for current smokers, although most interviewees also said they would participate. Attending lung cancer screening was likened to “facing the music” (P18, M, S, 47), and even discussing the offer with a friend was perceived as difficult, with one participant avoiding even the word cancer, referring instead to “the big C” (P12, M, S, 68). Avoidance was commonly explored through the third person, perhaps due to social desirability, with participants describing instances of friends or family avoiding seeking medical help for lung cancer symptoms until they were unwell (see Table 4).

#### 3.10.4 Too late: smoking damage and older age

The perception that irreparable smoking damage had already been done was common and related to the belief that it is too late to prevent lung cancer or change the course of their respiratory health. Smokers in particular discussed how the “damage is already done” (P3, M, S, 63) and appeared not to see screening as an opportunity to regain control over their risk of lung cancer mortality because “once you've smoked more than 10 years you think if I've got it, there's nothing I can do” (P4, F, X, 58). One ex‐smoker described the process of seeing smoking damage inside the lungs as “like going to a mortuary” (P1, M, X, 54).

Tellingly, while most thought that screening should be available to all, some believed it would be of greatest benefit to younger adults, due to their shorter smoking history and ability to “withstand any treatment” (P23, M, X, 66). The benefit of screening for older smokers was described as relatively low, which is contradictory to the screening eligibility criteria. Related to this, some participants attributed their risk of lung cancer, and life expectancy more generally, to chance (see Table 4).

#### 3.10.5 Screening: approach and support

Participants emphasized the need to provide a non‐judgemental screening service and suggested phrasing the invitation in general terms (i.e. in relation to an age group not smoking status), to normalize the offer. Others warned mentioning smoking cessation would be off‐putting to smokers and unnecessary, because this advice would be expected.

Some thought there should be interpersonal support preceding and during the appointment including “a family friend that could support them” (P17, F, S, 61), “a chat beforehand just to basically find out what… the procedure entails” (P7, F, X, 57) and “a phone number they could ring” (P4, F, X, 58). Others noted that the invitation wording should be considered carefully so as not to imply lung cancer is specifically suspected (see Table 4).

## 4 Discussion

To our knowledge, this is the first study to explore attitudes towards lung cancer screening among smokers and ex‐smokers from socioeconomically deprived communities using proactive recruitment methods to engage a “hard‐to‐reach” sample. The majority were superficially supportive of screening, but interviewee accounts were often contradictory, acknowledging early detection could be beneficial, but alongside negative views of treatment and survival, especially for older smokers. The findings gave insight into the salience of fear, avoidance, fatalism and stigma around lung cancer screening, particularly for smokers (as opposed to former and never smokers) which could deter participation.

The level of fear of lung cancer is consistent with studies describing higher cancer fear among low SES groups.^36^ Fear discussed during the interviews most commonly concerned an expected diagnosis at screening due to acknowledgement that a significant smoking history put them at high risk. Survey data showed that this fear was substantially more common among smokers. Almost three‐quarters agreed that they often worry about their chance of getting lung cancer, compared with a quarter of former and never smokers, an interestingly similar proportion given that former smokers are at greater risk than never smokers. Furthermore, a preference “not to know” was implicated in smokers’ avoidance of screening by interviewees. Cancer fear has been shown to promote and deter screening uptake in other programmes,^37^ but worry about what the doctor might find is a commonly reported reason by smokers for delaying symptomatic presentation.^38^ Therefore, emphasizing risk in screening communication could be counterproductive and unnecessary. Attempts to target risk perceptions using written information prior to screening have been unfruitful, with some evidence this strategy polarizes risk perceptions.^39^ Also, most smokers perceive their risk of lung cancer as higher than never smokers,^40^ with over a third we surveyed agreeing that their risk was higher than other smokers, and close to one‐fifth thinking they may already have lung cancer, more than former and never smokers. In a US study,^41^ smokers’ awareness of their increased risk was not associated with screening interest, and in the UKLS trial, higher affective risk perceptions were associated with non‐participation.^26^ Research should explore the conditions under which perceived risk can motivate and demotivate screening attendance among smokers and unpick the constituents of lung cancer fear.

To compound fear of diagnosis, smokers in particular believed that it was too late to benefit from screening due to irreversible smoking damage, despite being told the eligible age bracket. When interviewed, both current and former smokers described fatalistic perceptions, seeing lung cancer as an uncontrollable disease for which treatment was ineffective and survival was poor. The survey data suggested that smokers more commonly held fatalistic beliefs than their non‐smoking counterparts: around half perceived lung cancer to be a death sentence. Exploration during the interviews suggested this may stem from vicarious experiences of suffering with lung cancer, feeling entrapped by tobacco addiction and a general pessimism fostered by poor life circumstances. Of particular note was the perception that the lungs are not treatable for cancer because they are a vital organ that cannot be removed. Indeed, over a fifth of survey participants believed that most treatment is actually worse than lung cancer. Negative views of, and low confidence in, treatment could be instrumental in deterring screening participation by undermining the extent to which early diagnosis can be perceived as beneficial. The significance of worry, avoidance and fatalism to engagement with lung cancer screening has been observed previously,^27^ and fatalism towards cancer has been associated with avoidance of early detection^42^ and later stage at diagnosis.^43^ Unfortunately, a poor prognosis for lung cancer is the most common outcome due largely to late diagnosis,^3^ and it will be difficult to overlie this with the claim for benefits of early detection. A lay explanation for early stage treatment may help, one that addresses concerns and challenges the perception that the removal of a whole lung is necessary for treatment to be effective. This could be achieved by emphasizing the targeted nature of early treatment by showing the lungs subdivided into lobes.

Qualitative findings also gave insight into the roles that perceived stigma and tobacco dependence may play. Regret, guilt and feelings of entrapment by smoking were commonly expressed. The stigma attached to lung cancer as a self‐inflicted, smokers’ disease was especially apparent, which has been reported previously,^44^ and implicated as a deterrent to screening by a US study of long‐term smokers.^45^ Addiction is a disorder of motivation^46^ that could affect a person's confidence in their ability to quit and attitudes towards screening; perhaps by undermining perceived benefit. Similar observations of perceived blame and the expectation that treatment might be denied have been implicated in delayed symptomatic help‐seeking.^47^, ^48^, ^49^, ^50^ Non‐confrontational communication strategies that normalize the offer and reduce blame could improve engagement of more dependent smokers.

Limitations to the study design restrict our conclusions. By proactively recruiting in community settings outside of health service and trial contexts, we achieved a sample characterized by factors predictive of lower participation. However, we cannot assume the attitudes identified here are prevalent or generalizable beyond the English‐speaking urban communities we accessed, and further research should investigate the views of minority high‐risk groups. We also cannot infer that these attitudes differentiate lower and higher SES groups, because the latter were not recruited. While we report quantitative results separately by smoking group, our inclusion of screening‐ineligible adults (approaching the age threshold for eligibility and never smokers) may have biased our findings. In addition, it is possible that completing the survey primed the responses of the interviewees and that assisting those participants who needed help completing the questionnaire unintentionally biased their answers. Furthermore, the importance of these results for screening participation is speculative and large samples with representative sampling techniques and comprehensive measures are needed to quantify these findings. Studies are also needed that explore the association between these beliefs, screening intentions and ultimately screening behaviour. A randomized controlled trial^51^ is underway testing targeted invitation materials designed to improve uptake, which were informed by the results of this study.

In conclusion, attitudes towards lung cancer screening among lower SES smokers and former smokers are complex, but smokers appear to be the most negative when compared with former and never smokers. Many participants appeared to be superficially positive about screening and the benefits of early detection, but more detailed discussions identified feelings of loss of control of respiratory health and risk of lung cancer, fear and fatalism about diagnosis, blame and the belief that they would be unlikely to benefit personally. Addiction, difficult life circumstances and negative perceptions of treatment appeared to exacerbate this pessimism and lack of control. The approach of lung cancer screening is distinct in targeting individuals based on a highly stigmatized and widely publicized risk behaviour, and the expectation of a diagnosis among smokers appears to complicate decision‐making. To achieve an equitable and effective lung cancer screening programme, invitation strategies need to be evidence‐based and designed in consultation with low SES smokers to ensure they are sensitive to these complex attitudes.

## Conflict of Interest

AM has received travel funding, honorariums and consultancy payments from manufacturers of smoking cessation products (Pfizer Ltd, Novartis UK and GSK Consumer Healthcare Ltd) and hospitality from North51 who provide online and database services. AM also receives payment for providing training to smoking cessation specialists, receives royalties from books on smoking cessation and has a share in a patent of a nicotine delivery device. AM is an Associate of the New Nicotine Alliance (NNA) that works to foster greater understanding of safer nicotine products and technologies. SLQ, LAVM, SMJ and JW have no competing interests to declare.

## Sources of Funding

The study was funded by a National Awareness and Early Diagnosis Initiative (NAEDI) project grant awarded to JW (Principal Investigator) by Cancer Research UK and a consortium of funders (Department of Health (England); Economic and Social Research Council; Health and Social Care R&D Division, Public Health Agency, Northern Ireland; National Institute for Social Care and Health Research, Wales; Scottish Government). JW was, and LAVM and AM are, supported by Cancer Research UK. SLQ is supported by the Medical Research Council. SMJ is a Wellcome Trust Senior Fellow in Clinical Science and is supported by Rosetrees Trust, the Welton Trust, the Garfield Weston Trust and UCLH Charitable Foundation. This work was partially undertaken at UCLH/UCL who received a proportion of funding from the Department of Health's NIHR Biomedical Research Centre's funding scheme (SMJ). SMJ is funded by the Roy Castle Lung Cancer Foundation and is part of the CRUK Lung Cancer Centre of Excellence.

## Role of the Funder

Funders have had no role in the study design, the collection, analysis, interpretation of data, the writing of the article or the decision to submit it for publication.
