# Supplementary material for: Attitudes towards lung cancer screening in socioeconomically deprived and heavy smoking communities: informing screening communication
Source: Health Expect. 2016 Jul 11;20(4):563–73. doi: 10.1111/hex.12481 (PMC5513004; doi:10.1111/hex.12481)
Supplement: Supplementary file 1 [file HEX-20-563-s001.doc]

ONLINE SUPPLEMENT: Table of additional quantitative results

| Additional frequencies and univariate chi square analyses for agreement with each belief item by smoking status (Survey Study One) | | | | | | |  |
| --- | --- | --- | --- | --- | --- | --- | --- |
|  |  | | **Smoking status %(n) agree** | | | |  |
|  |  | | **Overall**  (n=163) | **Current**  (n=45) | **Former**  (n=71) | **Never**  (n=47) | **Sig.** |
| ***Perceived benefit of screening*** | | |  |  |  |  |  |
|  | | Lung cancer spreads so quickly that a CT scan would not help | 15.2 (24) | 22.7 (10) | 14.5 (10) | 8.9 (4) | .321^a^ |
|  | | A CT scan could improve my chances of surviving lung cancer | 64.8 (105) | 64.4 (29) | 71.4 (50) | 55.3 (26) | .386^a^ |
|  | | The treatment for lung cancer may not be as bad if the cancer is found early | 70.4 (112) | 75.0 (33) | 70.0 (49) | 66.7 (30) | .931^b^ |
|  | | I am too old to benefit from lung cancer screening | 12.4 (20) | 17.8 (8) | 11.4 (8) | 8.7 (4) | .080^b^ |
| ***Avoidance*** | | |  |  |  |  |  |
|  | | I avoid things that remind me of lung cancer | 24.7 (39) | 29.5 (13) | 21.7 (15) | 24.4 (11) | .866^b^ |
|  | | I avoid talking about lung cancer | 21.9 (35) | 31.1 (14) | 17.4 (12) | 19.6 (9) | .124^b^ |
|  | | I would not want to know if I have lung cancer | 18.4 (30) | 20.0 (9) | 14.1 (10) | 23.4 (11) | .560^b^ |
| ***Symptoms*** | | |  |  |  |  |  |
|  | | I do not need to have a lung cancer screening test because I have no symptoms | 19.6 (31) | 11.1 (5) | 17.4 (12) | 31.8 (14) | .111^b^ |
|  | | A symptom has to be severe for it to be a possible symptom of lung cancer | 11.8 (19) | 15.2 (7) | 8.6 (6) | 13.0 (6) | .475^a^ |
|  | | Lung cancer screening is only necessary if you have symptoms | 17.9 (29) | 20.0 (9) | 14.3 (10) | 21.3 (10) | .389^b^ |
|  | | People with lung cancer would have pain or other symptoms before being diagnosed | 32.7 (51) | 52.3 (23) | 19.1 (13) | 34.1 (15) | .009^a^ |
| ***Practicalities of screening*** | | |  |  |  |  |  |
|  | | I do not have the time to go for a lung cancer screening test | 5.0 (8) | 2.3 (1) | 4.3 (3) | 8.7 (4) | .389^b^ |
|  | | I have other priorities which are more important than getting a lung cancer screening test | 23.1 (37) | 24.4 (11) | 19.1 (13) | 27.7 (13) | .666^b^ |
|  | | Having to travel to my local hospital would keep me from having a lung cancer screening test | 10.7 (17) | 9.1 (4) | 9.9 (7) | 13.6 (6) | .729^b^ |
|  | | Having a CT scan exposes me to unnecessary radiation | 10.8 (17) | 11.4 (5) | 11.8 (8) | 8.9 (4) | .986^b^ |

NOTE: ^a^ χ²; ^b^ Fisher’s exact test

|  | | | | | |  |
| --- | --- | --- | --- | --- | --- | --- |
|  |  |  | | | |  |
|  |  |  |  |  |  |  |
|  | |  |  |  |  |  |
|  |  |  |  |  |  |  |
|  |  |  |  |  |  |  |
|  |  |  |  |  |  |  |
|  |  |  |  |  |  |  |
|  | |  |  |  |  |  |
|  |  |  |  |  |  |  |
|  |  |  |  |  |  |  |
|  |  |  |  |  |  |  |
|  | |  |  |  |  |  |
|  |  |  |  |  |  |  |
|  |  |  |  |  |  |  |
|  |  |  |  |  |  |  |
|  |  |  |  |  |  |  |
|  | |  |  |  |  |  |
|  |  |  |  |  |  |  |
|  |  |  |  |  |  |  |
|  |  |  |  |  |  |  |
|  |  |  |  |  |  |  |
